# Rapid Detection of Polychlorinated Biphenyls at Trace Levels in Real Environmental Samples by Surface-Enhanced Raman Scattering

**DOI:** 10.3390/s111110851

**Published:** 2011-11-18

**Authors:** Qin Zhou, Xian Zhang, Yu Huang, Zhengcao Li, Zhengjun Zhang

**Affiliations:** 1 Advanced Materials Laboratory, Department of Materials Science and Engineering, Tsinghua University, 30 Shuangqing Road, Beijing 100084, China; E-Mails: qinzhou@tsinghua.edu.cn (Q.Z.); xian-zhang09@mails.tsinghua.edu.cn (X.Z.); huangyu0818@163.com (Y.H.); zcli@tsinghua.edu.cn (Z.L.); 2 Institute of Nuclear and New Energy Technology, Tsinghua University, 30 Shuangqing Road, Beijing 100084, China

**Keywords:** SERS, polychlorinated biphenyls, trace amount, silver nanorods

## Abstract

Detection of trace levels of persistent pollutants in the environment is difficult but significant. Organic pollutant homologues, due to their similar physical and chemical properties, are even more difficult to distinguish, especially in trace amounts. We report here a simple method to detect polychlorinated biphenyls (PCBs) in soil and distilled spirit samples by the surface-enhanced Raman scattering technique using Ag nanorod arrays as substrates. By this method, polychlorinated biphenyls can be detected to a concentration of 5 μg/g in dry soil samples within 1 minute. Furthermore, based on simulation and understanding of the Raman characteristics of PCBs, we recognized homologues of tetrachlorobiphenyl by using the surface-enhance Raman scattering method even in trace amounts in acetone solutions, and their characteristic Raman peaks still can be distinguished at a concentration of 10^−6^ mol/L. This study provides a fast, simple and sensitive method for the detection and recognition of organic pollutants such as polychlorinated biphenyls.

## 1. Introduction

Persistent organic pollutants (POPs), such as dioxins, and polyclorinated biphenyls (PCBs), *etc*. are harmful to human health, and have polluted almost everywhere in the World [1]. In recent years, great research interest in the removal of these pollutants has been aroused, in which techniques that are able to detect these compounds even at trace levels play an important part. This is because they can accumulate to a high dose in human bodies through the food chain (vegetables, plants, animals, *etc.*) and cause severe diseases when the exceeds the critical dose, even when they are at trace levels in the environment [1–3]. Currently, the combination of high-resolution gas chromatography (GC) and mass spectrometry (MS) is widely used as a powerful means for the detection of these compounds. However, the GC/MS method is expensive and time-consuming, and is often not able to distinguish homologues [4–7].

Nanostructured materials exhibit many interesting properties and may find use in the detection of trace amounts of organic pollutants [8–11]. For example, some organics were detected by Surface-Enhanced Raman Scattering (SERS) in trace amounts, using noble metal (Cu, Ag and Au) nanostructures as the substrate [12–18]. SERS is a high sensitivity, simple, fast detection technique, that is also capable of recognition of compounds. Therefore investigating the possibility of using SERS in detection/recognition of POPs such as PCBs is of great interest [19,20]. The main problem of using SERS for PCBs is that they are insoluble in water, the solvent normally used in SERS measurements [21,22].

Here, we report first our study on the Raman spectra of polyclorinated biphenyl homologues both experimentally and theoretically, from which trace amount polyclorinated biphenyls in dry soil samples and distilled spirit can be detected and trace levels of homologues of tetrachlorobiphenyl in acetone can be distinguished via the SERS technique.

## 2. Experiments

Polluted soil samples which were acquired from the Nanjing Institute of Soil in China were dried and converted into fine powders. With the combination of high-resolution gas chromatography and mass spectrometry technique, sample I was shown to contain about 5 μg/g PCBs, and the sample II contained about 300 μg/g PCBs. Soil sample I (0.2 g) was put into acetone (20 mL), and agitated uniformly for about 5 minutes. This suspension was precipitated for 30 minutes, and the transparent acetone solution in the upper layer was taken as solution sample A. Next soil sample I (0.2 g) was put into acetone (200 mL), and solution sample B was obtained through the aforementioned process. Soil sample II (0.2 g) was put into acetone (20 mL) to obtain solution sample C and into acetone (200 mL) to obtain solution sample D.

**Figure 1.**
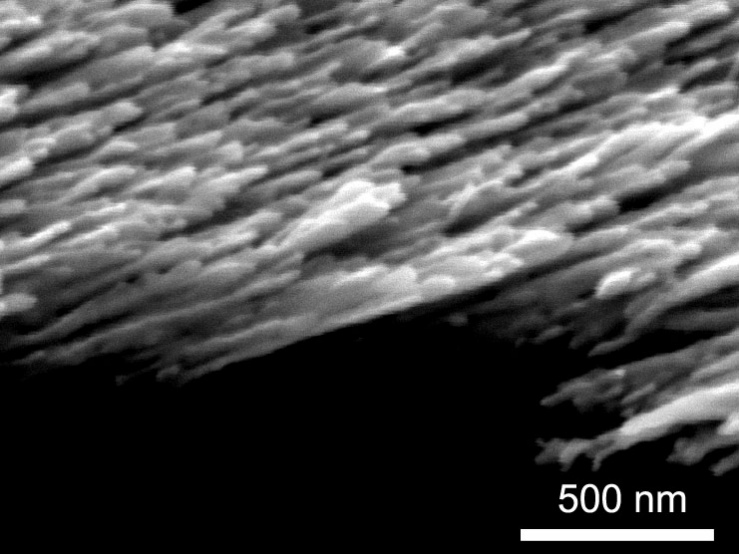
A typical SEM image showing the morphology of Ag nanorods used as the SERS substrates in this study.

The sensitivity of SERS substrates plays an important part in SERS measurements, and nanostructured noble metals were found to be high sensitive. In our study, we chose Ag nanorods as SERS substrates. They were prepared by electron beam deposition via the glancing angle deposition method. Details of the deposition of the Ag nanorods are described elsewhere [23]. The morphology of the Ag nanorods substrates is shown in Figure 1. The Ag nanorods are 30∼40 nm in diameter, and 600∼800 nm in length. The Ag nanorods SERS substrates were put into the solution samples A, B, C and D, respectively. After 30 minutes, the Ag nanorod substrates were taken out of the solutions and acetone on the substrates was blown away using a nitrogen flow. The Raman spectra of these substrates dipped into solution samples were measured at room temperature by a Renishaw Raman 100 spectrometer using a 633 nm He-Ne laser as the excitation source.

## 3. Results and Discussion

### 3.1. Detect PCBs at Trace Amount in Soil by SERS

**Figure 2.**
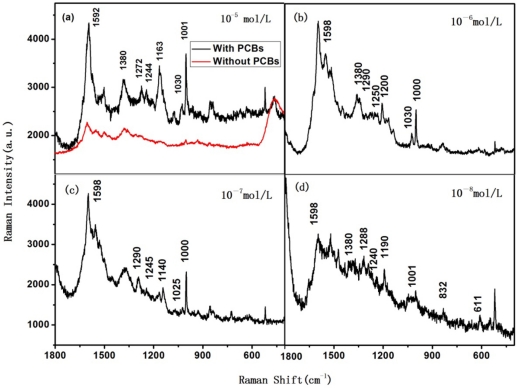
Raman spectra of PCBs polluted soil samples with PCBs concentration of (**a**) 10^−5^ mol/L and without PCBs for comparison; (**b**) 10^−6^ mol/L; (**c**) 10^−7^ mol/L; (**d**) 10^−8^ mol/L.

Figures 2(a–d) show the measured Raman spectra of the Ag substrates dipped into samples A, B, C and D, respectively. Soil without PCBs acquired from the Nanjing Institute of Soil was also treated as described before, and the Raman spectrum of the Ag substrate dipped into this sample was also shown in Figure 2a for comparison (red trace). From Figures 2(a–c), one clearly sees peaks at ∼1,600, 1,280, 1,240, 1,150, 1,030 and 1,000 cm^−1^, demonstrating the common feature of PCBs. The peaks around 1,590∼1,600 cm^−1^ represent the benzene bending vibration mode; the peak around 1280 cm^−1^ represents the CC bridge bending vibration mode; the peak around 1030 cm^−1^ represents the CH bending in-plane mode; the peak around 1,000 cm^−1^ represents the trigonal breathing vibration mode; and peaks around 1,240∼1,250 cm^−1^ and 1,140∼1,200 cm^−1^ represent the vibration peaks induced by the Cl substituents [24–27]. These characteristic peaks suggest that PCBs in dry soil can be detected by the SERS method by dissolving into acetone. The most widely used PCBs are trichlorobiphenyls and pentachlorobiphenyls, so we assumed that the molecular weight of the PCBs in the soil samples is 300, then the concentration of the PCBs acetone solution in solution sample A, B, C and D are about 10^−5^ mol/L, 10^−6^ mol/L, 10^−7^ mol/L, and 10^−8^ mol/L, respectively. As the characteristic peaks in Figure 2(d) are not clear, the detection limit in our study is about 5 μg/g. Therefore, as the contents in the soil samples are complicated, we are not capable to distinguish other contents via Raman peaks in our study.

### 3.2. Detect PCBs in Distilled Spirit by SERS

PCBs in distilled spirit can also be detected by the SERS method with silver nanorod substrates. The concentration of PCBs in distilled spirit is about 10^−4^ mol/L.

**Figure 3.**
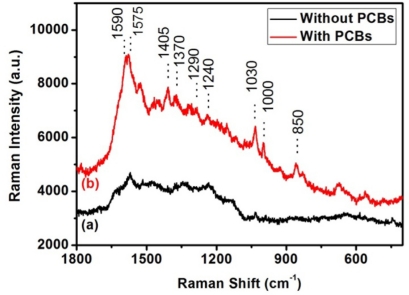
Raman spectra of curve (**a**) Pure distilled spirit; (**b**) Distilled spirit with 10^−4^ mol/L PCBs.

We put a drop of PCB-“polluted” distilled spirit on the silver nanorod substrates and volatilized away the distilled spirit. Then, we found PCB Raman signal with the SERS method described before. Figure 3 curves (a) and (b) show the SERS spectra of pure distilled spirit and distilled spirit with 10^−4^ mol/L PCBs, respectively. One can recognize the characteristic Raman peaks of PCBs around 1,590, 1,290, 1,240, 1,030 and 1,000 cm^−1^ in Figure 3, curve (b), which suggests the presence of PCBs.

### 3.3. Recognize Tetrachlorobiphenyl at Trace Amount by SERS

One may notice that, although trace amount PCBs in dry soil can be detected via the SERS technique, to distinguish PCBs from their 209 isomers and homologues is still a problem. Indeed, isomers and homologues of organic pollutants have similar physical and chemical properties, thus, they are hard to distinguish, especially in trace amounts. As different isomers and homologues have different vibration modes, their Raman shifts correspond to these vibration mode are different, too. By this way, the SERS method with silver nanorods as a substrate can also be used to identify the Raman characteristics of isomers of PCBs, even at trace levels.

The Raman spectra of 2,2′,3,3′-tetrachlorobiphenyl, 2,2′,6,6′-tetrachlorobiphenyl, 2,3,5,6-tetrachlorobiphenyl, and 3,3′,5,5′-tetrachlorobiphenyl were measured by a Renishaw Raman 100 spectrometer using a 633 nm He-Ne laser as the excitation source at room temperature. Powders of these compounds are commercially available from the AccuStandard Company (New Haven, CT, USA). Simulation of these Raman spectra was performed using the Gaussian 03 programme package using the density functional theory, to better understand the observed vibrational modes and figure out the fingerprints of these compounds. For the SERS measurements, chlorobiphenyl powders were dissolved in acetone to concentrations ranging from 10^−4^ to 10^−10^ mol/L. The substrates were Ag nanorods prepared by electron beam deposition. The deposition of the Ag nanorods was as described before. A small volume of the solutions (∼0.5 μL) was dropped on the surface of Ag nanorods and acetone was blown away using a nitrogen flow.

To gain a clear understanding of these features, we performed simulations using the Gaussian 03 programme package using density functional theory. The simulations were carried out with Becke’s three-parameter hybrid method using the Lee-yang-Parr correlation functional (B3LYP) and the LANL2DZ basis set [28]. Gaussian View was used to input investigated compounds’ data visually. The bond length of the benzene ring was set to be 1.409 Å, the π bond length between C and H atoms was set to be 1.088 Å and the σ bond length between C and Cl atoms was set to be 1.760 Å.

According to the simulation, we can recognize the isomers of tetrachlorobiphenyl at trace levels through the following Raman peaks corresponding to C-H vibration mode: 2,2′,3,3′-tetrachlorobiphenyl and 2,2′,6,6′-tetrachlorobiphenyl show strong peaks around 490 cm^−1^, which correspond to C-H bending vibration mode; the same peak of 2,3,5,6-tetrachlorobiphenyl is around 640 cm^−1^, and for 3,3′,5,5′-tetrachlorobiphenyl it is around 410 cm^−1^. From these peaks, one can tell 2,3,5,6-tetrachlorobiphenyl and 3,3′,5,5′-tetrachlorobiphenyl from the four tetrachlorobiphenyl isomers.

As the C-H bending in-plane energy of 2,2′,3,3′-tetrachlorobiphenyl is higher than that of 2,2′,6,6′-tetrachlorobiphenyl, the strongest C-H bending in-plane peak of the former is around 1,150 cm^−1^ (between 1,250 and 1,100 cm^−1^), while the one of the latter is around 1,100 cm^−1^. In this way, one can tell 2,2′,3,3′-tetrachlorobiphenyl from 2,2′,6,6′-tetrachlorobiphenyl via the corresponding SERS spectrum.

Besides the above characteristic peaks, the different substituents also influence the C-C bridge stretching vibration energy. C-C bridge bond stretching makes 3,5-substituent Cl atoms move, thus the C-C bridge stretching peak of 2,2′,6,6′-tetrachlorobiphenyl is relatively small, and is around 1,260 cm^−1^; while this peak in 2,2′,3,3′-tetrachlorobiphenyl and 3,3′,5,5′-tetrachlorobiphenyl is between 1,275 and 1,295 cm^−1^.

**Figure 4.**
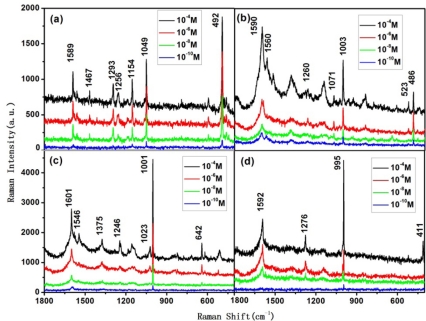
SERS spectra of tetrachlorobiphenyls at concentrations from 10^−4^ to 10^−10^ mol/L in acetone (**a**) 2,2′,3,3′-tetrachlorobiphenyl; (**b**) 2,2′,6,6′-tetrachlorobiphenyl; (**c**) 2,3,5,6-tetrachlorobiphenyl; (**d**) 3,3′’,5,5′-tetrachlorobiphenyl.

Figures 4(a–d) show the Raman spectra of 10^−4^ to 10^−10^ mol/L 2,2′,3,3′-tetrachlorobiphenyl, 2,2′,6,6′-tetrachlorobiphenyl, 2,3,5,6-tetrachlorobiphenyl, and 3,3′,5,5′-tetrachlorobiphenyl, respectively. The characteristic peaks mentioned above remain visible at 10^−6^ mol/L, thus one can recognize each one of these four isomers via the aforementioned method, even at concentration of 10^−^^6^ mol/L.

## 4. Conclusions

In summary, we have demonstrated here a simple method to detect PCBs dry soil and distilled spirit samples, even in trace amounts, by using the SERS technique with Ag nanorods as substrates. We also recognized isomers of tetrachlorobiphenyls at trace levels based on the understanding of the Raman characteristics of these compounds. This method might be applicable to the detection of PCBs, which is crucial for the removal of these harmful hazardous pollutants from the environment.
